# Differential Effects of apoE4 and Activation of ABCA1 on Brain and Plasma Lipoproteins

**DOI:** 10.1371/journal.pone.0166195

**Published:** 2016-11-08

**Authors:** Anat Boehm-Cagan, Roni Bar, Dror Harats, Aviv Shaish, Hana Levkovitz, John K. Bielicki, Jan O. Johansson, Daniel M. Michaelson

**Affiliations:** 1 The Department of Neurobiology, The George S. Wise Faculty of Life Sciences, The Sagol School of Neuroscience, Tel Aviv University, Tel Aviv 6997801, Israel; 2 Sackler Faculty of Medicine, Tel-Aviv University, Tel Aviv 6997801, Israel; 3 The Bert W. Strassburger Lipid Center, Sheba Medical Center, Tel-Hashomer 5265601, Israel; 4 Life Sciences Division, Lawrence Berkeley National Laboratory, University of California, Berkeley, California, 94720, United States of America; 5 Artery Therapeutics, Inc. San Ramon, California, United States of America; Torrey Pines Institute for Molecular Studies, UNITED STATES

## Abstract

Apolipoprotein E4 (apoE4), the leading genetic risk factor for Alzheimer's disease (AD), is less lipidated compared to the most common and AD-benign allele, apoE3. We have recently shown that i.p. injections of the ATP-binding cassette A1 (ABCA1) agonist peptide CS-6253 to apoE mice reverse the hypolipidation of apoE4 and the associated brain pathology and behavioral deficits. While in the brain apoE is the main cholesterol transporter, in the periphery apoE and apoA-I both serve as the major cholesterol transporters. We presently investigated the extent to which apoE genotype and CS-6253 treatment to apoE3 and apoE4-targeted replacement mice affects the plasma levels and lipid particle distribution of apoE, and those of plasma and brain apoA-I and apoJ. This revealed that plasma levels of apoE4 were lower and eluted faster following FPLC than plasma apoE3. Treatment with CS-6253 increased the levels of plasma apoE4 and rendered the elution profile of apoE4 similar to that of apoE3. Similarly, the levels of plasma apoA-I were lower in the apoE4 mice compared to apoE3 mice, and this effect was partially reversed by CS-6253. Conversely, the levels of apoA-I in the brain which were higher in the apoE4 mice, were unaffected by CS-6253. The plasma levels of apoJ were higher in apoE4 mice than apoE3 mice and this effect was abolished by CS-6253. Similar but less pronounced effects were obtained in the brain. In conclusion, these results suggest that apoE4 affects the levels of apoA-I and apoJ and that the anti-apoE4 beneficial effects of CS-6253 may be related to both central and peripheral mechanisms.

## Introduction

Apolipoprotein E (apoE) is the most abundant lipoprotein in the brain, where it is produced mostly by astrocytes [1]. ApoE is also synthesized in the periphery, typically by the liver and by macrophages [2], and is an important serum lipoprotein. ApoE exists as three major isoforms, termed apoE2, apoE3 and apoE4, of which apoE4 is the most prevalent genetic risk factor for Alzheimer's disease (AD) [3–5].

Studies performed in humans and corresponding mouse models which express apoE4, suggest that brain apoE4 is less lipidated compared to apoE3, the most common and AD-benign apoE allele [6–9]. These findings are supported by *in vitro* cell culture experiments which revealed that apoE4 is less effective than apoE3 in promoting cholesterol and phospholipid efflux [10–12]. ApoE lipidation is achieved by the ATP-binding cassette transporters A1 and G1 (ABCA1 and ABCG1, respectively), which stimulate apolipoprotein lipidation both in the brain and the periphery [13, 14]. CSF samples extracted from AD patients have lower ex-vivo capacity for ABCA1-mediated cholesterol efflux compared to controls, an effect which is exacerbated in apoE4 carriers [15]. In the periphery, the main function of ABCA1 is to transport cholesterol and phospholipids from the plasma membrane of peripheral cells to lipid-free or lipid-poor apolipoprotein, such as apoE and apoA-I. This process which initiates the formation of high density lipoproteins (HDL) is called reverse cholesterol transport [16]. In the brain, ABAC1 stimulates the lipidation of apoE following its synthesis and secretion from astrocytes [17]. Interestingly, brain and peripheral apoE are produced and regulated independently and are, thus, parts of separate metabolic pools which do not mix [18, 19].

ApoA-I is the major apolipoprotein of HDL in the periphery, where it plays an important role in reverse cholesterol transport. ApoA-I, though not synthesized in the brain, is present in the central nervous system (CNS), due to its ability to cross the blood brain barrier (BBB) from the periphery [19, 20]. In view of the important role of apoA-I in lipid transport in the periphery, it is presumed that apoA-I plays a similar role in the brain [20–22]. Clusterin (apoJ) is another major apolipoprotein in the brain. It is less lipidated than apoE [23], and is believed to play a role in lipid transport and as a molecular chaperon associated with stress response [13, 24].

We have recently shown, utilizing apoE-targeted replacement (TR) mice, that direct activation of the lipidating protein ABCA1 using intraparietal (i.p.) injections of the ABCA1-agonist peptide CS-6253 results in the accumulation of the peptide in the brain and in reversal of the hypolipidation of brain apoE4 which is observed in the non-treated mice [25]. Importantly, these effects were associated with reversal of the brain pathological effects of apoE4 and of the corresponding apoE4-driven cognitive impairments [25].

Since the protective anti-apoE4 effects of the ABCA1 agonist CS-6253 were observed following the peripheral application of this peptide and in view of the important role of ABCA1 in mediating the lipidation of apoE and apoA-I, we presently examined the extent to which the neuroprotective effects of the CS-6253 may be related to changes in the levels and extent of lipidation of peripheral apoE4 and/or to corresponding changes in the levels and lipidation of apoA-I and apoJ in the plasma and in the brain.

## Materials and Methods

### Mice

ApoE-TR mice, in which the endogenous mouse apoE was replaced by either human apoE3 or apoE4, were created by gene targeting as previously described [26]. The mice used were homozygous for the apoE3 (3/3) or apoE4 (4/4) alleles, and were purchased from Taconic, who back-crossed them to wild-type C57BL/6J mice (2BL/ 610; Harlan Laboratories) for 7 generations. The mice were further back-crossed in our lab for 3 additional generations with wild-type C57BL/6J mice (2BL/ 610; Harlan Laboratories). These mice are referred to herein as apoE3 and apoE4 mice, respectively. The apoE genotype of the mice was confirmed by PCR analysis, as described previously [27, 28]. The mice were kept on standard chow-diet. All experiments were performed on age-matched male animals (4 months of age) and were approved by the Tel Aviv University Animal Care Committee. Every effort was made to reduce animal stress and to minimize animal usage. CS-6253 was kindly provided by Artery Therapeutics, Inc. and was administered according to a previously described *in vivo* protocol [29]. Accordingly, CS-6253 was injected intraperitoneally (i.p.) to 2.5-month-old male mice for 6 weeks (20mg/kg/48h, which translates to 0.5mg dissolved in 400μl of PBS per injection of a mouse weighing 25 grams). Corresponding control mice were injected with PBS (P5493, Sigma) in a similar manner. The mice were anesthetized with ketamine and xylazine, following termination of treatment, after which blood samples were collected and the mice were perfused transcardially with PBS. The brains were then removed from the PBS perfused mice and further processed for biochemical analysis, as outlined in the succeeding paragraphs.

### Preparation of plasma and brain samples

1. Plasma samples. Freshly excised blood was drawn from the posterior vena cava of the anesthetized mice and collected into tubes containing 20μl of 10% EDTA to prevent blood clotting. The blood was centrifuged for 10 min at 3000 rpm at 4°C, after which the supernatant containing the plasma lipoproteins was collected and frozen at -70°C until used.
2. Hippocampal homogenates. Hippocampus was rapidly removed from one freshly excised brain hemisphere and rapidly frozen in liquid nitrogen after which it was stored frozen at −70°C until used. Frozen hippocampi were thawed, homogenized with a Teflon-glass homogenizer at 4°C with cold Tris-buffered saline (TBS) containing protease inhibitor mixture (P8340; Sigma), and phosphatase inhibitor mixture (P5726; Sigma). The homogenized hippocampi were then aliquoted and rapidly frozen in liquid nitrogen after which they were kept at -70°C until used.

### Immunoblots

1. SDS-electrophoresis. The hippocampal extracts and plasma samples were thawed and boiled for 10 min with 0.5% SDS and immunoblotted as previously described [30, 31]. Gels were then transferred to a nitrocellulose membrane and stained with either goat anti-apoE Ab (1:10,000 for both hippocampal and plasma samples; Millipore), rabbit anti-apoA-I Ab (1:1000 for hippocampal homogenates and 1:5000 for plasma samples; Meridian Life Science, Inc.), and goat anti-apoJ Ab (1:5000 for hippocampal homogenates and 1:7000 for plasma samples; Santa Cruz). The immunoblot bands were all visualized using the ECL chemiluminescent substrate (Pierce), after which their intensity was visualized and quantified utilizing Image Lab software (Bio-Rad). GAPDH levels (mouse anti-GAPDH, 1:1000; Abcam) were used as gel-loading controls and the results are presented relative to the control apoE3 mice.
2. Blue native gels. This was performed with the hippocampal extracts, whereas the plasma samples were subjected to fast protein liquid chromatography (FPLC) as described below. Accordingly, the hippocampal homogenates were electrophoresed on 4–16% gels in the Native PAGE Novex Bis-Tris Gel System purchased from Novex and according to the manufacturer's instructions, as previously described [7, 25]. Gels were next transferred to PVDF membranes and stained with either goat anti-apoE Ab (1:10,000; Millipore), rabbit anti-apoA-I Ab (1:1000; Meridian Life Science, Inc.), or goat anti-apoJ Ab (1:1000; Santa Cruz). The immunoblot bands were all visualized using the ECL chemiluminescent substrate (Pierce).

### Fast protein liquid chromatography (FPLC) and cholesterol and triglycerides measurements

Total plasma cholesterol and triglycerides were measured using commercial kits (Chol, Roche/ Hitachi, Roche Diagnostics; Infinity, Thermo Electron). For FPLC, four samples from each mouse group were pooled together to generate 200μl of plasma extract, which was loaded and subjected to high resolution size exclusion/fast protein liquid chromatography (FPLC) using a Superose 6 column (Amersham Pharmacia Biotech AB, Piscataway, NJ), as previously described [32]. This process was performed twice and the results presented are the pool of the two columns performed for each group, as was determined by immunoblotting of the different FPLC fractions utilizing the appropriate anti-apolipoprotein Ab.

### Statistical analysis

The experimental design consisted of two genotypes (apoE3 and apoE4) and two treatments (control and CS-6253) and the results were analyzed using 2-way ANOVA testing with STATISTICA software (version 8.0; StatSoft). Only after 2-way ANOVA retrieved significant results, further *post hoc* Fisher analysis was performed to test for individual effects, and these findings are depicted in the figures. The results presented correspond to the mean ± SEM, and are normalized relative to control apoE3 mice. Each of the four groups contained n = 8–10 mice for apoJ, apoE and cholesterol analysis, and n = 5 mice for apoA-I analysis. For FPLC analysis, the fractions from each column were run in a western blot, and the area under the emerging curve was calculated and is depicted beneath the curve.

## Results

### 1. The effect of CS-6253 and the apoE genotype on plasma lipoproteins

#### 1.1. ApoE

**Fig 1:**
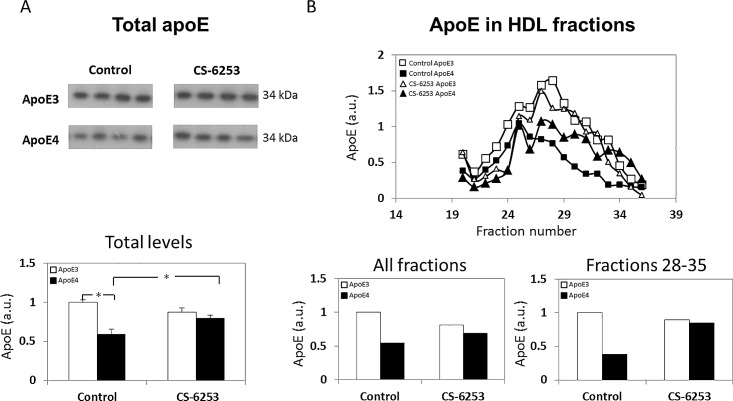
ApoE genotype and CS-6253 treatment affect plasma apoE levels and distribution in apoE3 and apoE4 mice. *(A) Total apoE levels*. ApoE3 and apoE4 mice were injected i.p. with CS-6253 or PBS and their plasma was subjected to western blot analysis utilizing a pan anti-apoE Ab, as described in the Materials and Methods. Representative immunoblots of four mice per group are presented on the upper panel, whereas quantitation of the results (mean ± SEM; n = 8–10 per group) normalized relative to control apoE3 mice is presented on the bottom panel. White bars correspond to apoE3 mice, whereas black bars correspond to apoE4 mice. Two-way ANOVA revealed a significant effect for genotype x treatment (p = 0.002). *p<0.*0001* for the post hoc comparison of control apoE4 and control apoE3 mice, and *p<0.*01* for the post hoc comparison of control apoE4 mice and CS-6253-treated apoE4 mice. *(B) Size distribution of apoE particles following FPLC*. Plasma samples from four mice per group (i.e., control and CS-6253 treated apoE3 and apoE4 mice) were pooled together and subjected to FPLC analysis after which the apoE content of the different fractions was determined by immunoblot measurements, as described in Materials and Methods. The distribution of apoE particles following FPLC is depicted in the upper panel: □ and ■ correspond to control apoE3 and apoE4 mice, respectively, whereas △ and ▲ correspond to CS-6253-treated apoE3 and apoE4 mice, respectively. Quantification of the results (*n* = 8 per group) in terms of the total amount of plasma apoE collected from all FPLC fractions of apoE4 mice (black bars) and apoE3 mice (white bars) are presented on the left bottom panel. Quantitation of the results obtained from fractions 28–35 is depicted on the bottom right panel.

The effects of apoE genotype and CS-6253 on the plasma levels of apoE and on the levels and type of lipid particles with which apoE is associated were assessed by SDS gel electrophoresis and FPLC. As shown in Fig 1, the total levels of apoE in the plasma were lower in the control apoE4 mice compared to the corresponding apoE3 mice (1.00 ± 0.03 versus 0.59 ± 0.06 for control apoE3 and control apoE4 mice, respectively). This effect was virtually abolished by the CS-6253 treatment, which elevated the levels of apoE in the treated-apoE4 mice while having no effect on the corresponding plasma level of the CS-6253-treated apoE3 mice (0.87 ± 0.05 versus 0.80 ± 0.03 for CS-6253-treated apoE3 and CS-6253-treated apoE4, respectively). Two-way ANOVA of these results revealed a significant effect of genotype x treatment (p = 0.02). Further *post hoc* analysis revealed that the levels of apoE were significantly lower in the control apoE4 mouse group compared to the control apoE3 mice (p<0.0001), and that the CS-6253 treatment significantly increased the levels of apoE in the apoE4-treated mice (p = 0.003). The extent to which this CS-6253-driven increase in plasma apoE4 is associated with changes in the size distribution of the apoE4 particles was assessed by FPLC, followed by apoE immunoblot analysis of the FPLC fractions. This revealed, in accordance with previous observations, that in the mouse plasma apoE elutes mostly as HDL particles [26, 33], and that the elution profile of plasma apoE was affected by both apoE genotype and CS-6253 (Fig 1B). Analysis of the total levels of apoE eluted from the column (i.e., the sum of apoE from all fractions) revealed a pattern similar to that obtained from the SDS analysis of total apoE levels in the plasma (compare Fig 1A and 1B). A marked difference also emerged in the elution profiles of apoE3 and apoE4, whereas apoE of the control apoE4 mice eluted from the column about 4 fractions earlier than the corresponding apoE3 mice, the CS-6253 treatment shifted the elution profile of apoE4 towards that of the apoE3 mice, which was not affected by this treatment. As the observed effect of CS-6253 was to shift the apoE4 peak towards the apoE3 peak, namely, towards fractions 28–35, the results were quantitated by focusing on these fractions (Fig 1B, right bottom panel). This revealed that the distinct decrease in apoE4 particles in this size range was markedly reversed by CS-6253. Taken together, these findings suggest that the total levels of plasma apoE are lower in the apoE4 mice compared to those of the apoE3 mice, and that CS-6253, which had no effect on plasma apoE3, had a pronounced effect on the plasma apoE4 particles, namely it shifted the apoE4 towards particles which elute later from the column. The mechanism underlying the finding that in the plasma CS-6253 seems to alter the plasma apoE4 particle (Fig 1B) will be addressed in the discussion.

#### 1.2. ApoA-I

**Fig 2:**
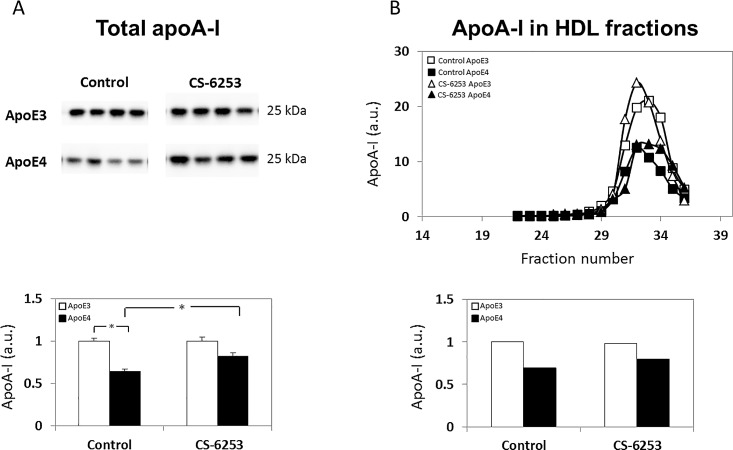
ApoE genotype and CS-6253 treatment affect plasma apoA-I levels and distribution in apoE3 and apoE4 mice. *(A) Total apoA-I levels*. ApoE3 and apoE4 mice were injected i.p. with CS-6253 or PBS, after which their plasma was subjected to western blot analysis with anti-apoA-I Ab, as described in the Materials and Methods. Representative immunoblots of four mice per group are presented on the upper panel, whereas quantitation of the results (mean ± SEM; n = 5 per group) normalized relative to control apoE3 mice is presented on the bottom panel. White bars correspond to apoE3 mice, whereas black bars correspond to apoE4 mice. Two-way ANOVA revealed a significant effect for genotype x treatment (p = 0.037). *p<0.*0001* for the post hoc comparison of control apoE4 and control apoE3 mice, and *p<0.*01* for the post hoc comparison of control apoE4 mice and CS-6253-treated apoE4 mice. *(B) Size distribution of apoA-I particles following FPLC*. Plasma samples from each of the four mice per group (i.e., control and CS-6253 treated apoE3 and apoE4 mice) were pooled together and subjected to FPLC analysis after which the apoA-I content of the different fractions was determined by immunoblot measurements, as described in the Materials and Methods. The apoA-I particle distribution following FPLC is depicted in the upper panel: □ and ■ correspond to control apoE3 and apoE4 mice, respectively, whereas △ and ▲ correspond to CS-6253-treated apoE3 and apoE4 mice, respectively. Quantification of the results (*n* = 8 per group) in terms of the total apoA-I collected from all FPLC fractions of apoE4 mice (black bars) and apoE3 mice (white bars) is presented on the bottom panel.

ApoA-I is a central HDL lipoprotein, and is a major substrate of ABCA1-induced lipidation in the periphery [34]. Therefore, we next examined the effects of ABCA1 activation and of apoE genotype on the levels and particle size distribution of plasma apoA-I. As can be seen in Fig 2A, the levels of apoA-I were lower in the control apoE4 mice compared to control apoE3 mice, and CS-6253 treatment increased the levels of apoA-I of the apoE4 mice to that of the apoE3 mice, without having an effect on the corresponding levels in the apoE3 mice (1.00 ± 0.04 versus 0.64 ± 0.03 for control apoE3 and control apoE4 mice, respectively, and 1.00 ± 0.05 versus 0.82 ± 0.03 for CS-6253-treated apoE3 and CS-6253-treated apoE4 mice, respectively). Two-way ANOVA of these results revealed a significant effect of genotype x treatment (p = 0.037), and further *post hoc* analysis revealed that the levels of apoA-I in the apoE4 control mice were significantly lower compared to those of the corresponding apoE3 mice (p<0.0001), and were significantly increased following treatment (p = 0.007). FPLC analysis revealed that the apoA-I fractions of the four mouse groups had a similar size distribution peaking at around fraction 33 (Fig 2B), which is later than the elution of the apoE particles (compare Figs 1B and 2B). Furthermore, and in accordance with the total measurements of apoA-I (Fig 2A), the levels of the FPLC-fractionated apoA-I were lower in the control apoE4 mice than those of the corresponding apoE3 mice, and this effect was partially reversed by CS-6253 treatment (Fig 2B).

#### 1.3. ApoJ

**Fig 3:**
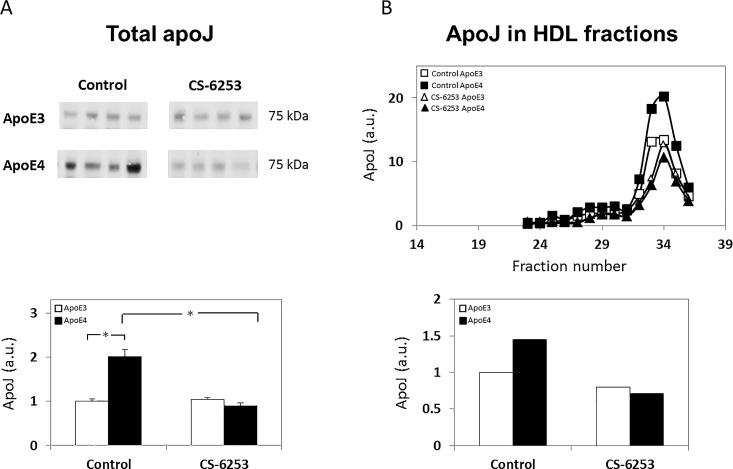
ApoE genotype and CS-6253 treatment affect plasma apoJ levels and distribution in apoE3 and apoE4 mice. *(A) Total apoJ levels*. ApoE3 and apoE4 mice were injected i.p. with CS-6253 or PBS, after which their plasma was subjected to western blot analysis with anti-apoJ Ab, as described in the Materials and Methods. Representative immunoblots of four mice per group are presented on the upper panel, whereas quantitation of the results (mean ± SEM; n = 8–10 per group) normalized relative to control apoE3 mice is presented on the bottom panel. White bars correspond to apoE3 mice, whereas black bars correspond to apoE4 mice. Two-way ANOVA revealed a significant effect for genotype x treatment (p<0.0001). *p<0.*0001* for the post hoc comparison of control apoE4 and control apoE3 mice, and *p<0.*0001* for the post hoc comparison of control apoE4 mice and CS-6253-treated apoE4 mice. *(B) Size distribution of apoJ particles following FPLC*. Plasma samples from four mice per group (i.e., control and CS-6253 treated apoE3 and apoE4 mice) were subjected to FPLC analysis after which the apoJ content of the different fractions was determined by immunoblot measurements, as described in Materials and Methods section. The distribution of apoJ particles following FPLC is depicted in the upper panel: □ and ■ correspond to control apoE3 and apoE4 mice, respectively, whereas △ and ▲ correspond to CS-6253-treated apoE3 and apoE4 mice, respectively. Quantification of the results (*n* = 8 per group) in terms of the total apoJ collected from all FPLC fractions of apoE4 mice (black bars) and apoE3 mice (white bars) is presented on the bottom panel.

We next examined the effects of apoE genotype and CS-6253 on plasma apoJ. As shown in Fig 3, the total levels of apoJ were significantly higher in the apoE4 control mice compared to the corresponding apoE3 mice (1.00 ± 0.05 versus 2.01 ± 0.16 for control apoE3 and control apoE4 mice, respectively). Importantly, this effect was completely abolished by CS-6253 treatment, which had no effect on the apoE3 mice (1.04 ± 0.04 versus 0.90 ± 0.05 for CS-6253-treated apoE3 and CS-6253-treated apoE4 mice, respectively). Two-way ANOVA revealed a significant genotype x treatment effect (p<0.0001). Further *post hoc* analysis revealed that the levels of apoJ were significantly higher in the control apoE4 mouse group compared to the control apoE3 mice (p<0.0001), and that the CS-6253 treatment significantly reduced the levels of apoJ in the apoE4-treated mice (p<0.0001). FPLC analysis revealed a similar pattern for all groups with a single peak climaxing at fraction 34. Quantitation of these FPLC results revealed, in accordance with the total apoJ measurements (Fig 3A), that they were higher in the control apoE4 mice relative to the corresponding apoE3 mice, and that this effect was abolished by CS-6253 (Fig 3B). The fact that the apoJ particles eluted later than the apoA-I and apoE particles (i.e., the apoJ peak was observed at fraction 34, while the apoE peak was observed at around fraction 28) is in accordance with previous findings that apoJ is associated with smaller lipoprotein particles compared to apoE and apoA-I [23, 35].

#### 1.4. Plasma cholesterol and triglycerides

**Fig 4:**
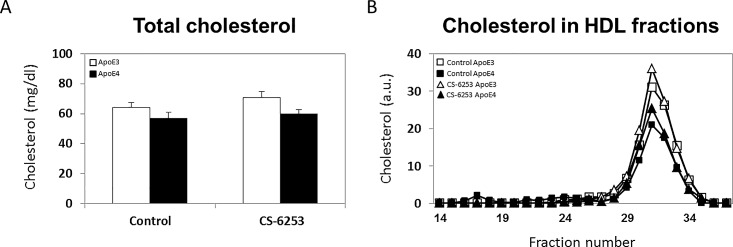
The effects of apoE genotype and CS-6253 on plasma cholesterol in apoE3 and apoE4 mice. ApoE3 and apoE4 mice were injected i.p. with CS-6253 or PBS, after which their total plasma cholesterol levels (A) and their cholesterol elution profile following *FPLC (B)* were determined, as described in the Materials and Methods. Results of the total cholesterol levels (mean ± SEM; *n* = 8–10 per group) are depicted as mg/dL. White bars correspond to apoE3 mice, whereas black bars correspond to apoE4 mice. Fig 4B depicts the cholesterol eluted from the FPLC column of the different mice groups (□ and ■ correspond to control apoE3 and apoE4 mice, respectively, whereas △ and ▲ correspond to CS-6253-treated apoE3 and apoE4 mice, respectively).

Measurements of the total plasma cholesterol levels revealed a small decrease in the apoE4 mice compared to apoE3 mice and that CS-6253 had no effect on the plasma cholesterol levels of both mouse groups (64.2 mg/dL ± 3.5 versus 57.0 mg/dL ± 3.9 for control apoE3 and control apoE4, respectively, and 70.9 mg/dL ± 4.0 versus 59.9 mg/dL ± 2.7 for CS-6253-treated apoE3 and CS-6253-treated apoE4 mice, respectively; see Fig 4). FPLC revealed that the cholesterol of the four mouse groups eluted as a single HDL-peak which co-eluted closely with apoA-I (peak at fractions 31–32 in Figs 2B and 4B), and whose levels, in accordance with the total cholesterol measurements, were somewhat lower in the apoE4 mice groups compared to the corresponding apoE3 mice (Fig 4B). Measurements of the total levels of triglycerides in the plasma revealed that like the cholesterol levels, they were slightly lower in the apoE4 control mice relative the control apoE3 mice (35.26 mg/dL ± 3.90 and 27.79 mg/dL ± 1.18 for the control apoE3 and control apoE4 mice, respectively), and that the triglycerides levels of both moue groups were not affected by CS-6253 (34.22 mg/dL ± 4.51 and 28.31 mg/dL ± 4.33 for the CS-6253-treated apoE3 and CS-6253-treated apoE4 mice, respectively).

### 2. The effect of CS-6253 and apoE genotype on brain lipoproteins

**Fig 5:**
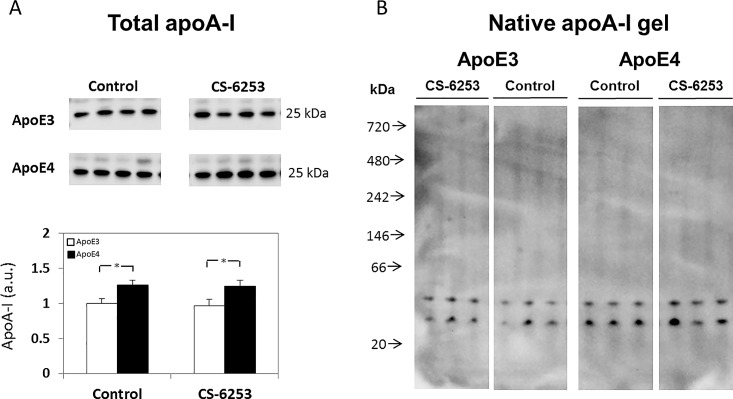
The effects of apoE genotype and CS-6253 on the levels and lipidation of brain apoA-I in apoE3 and apoE4 mice. *(A) Total apoA-I levels*. ApoE3 and apoE4 mice were injected i.p. with CS-6253 or PBS, after which their hippocampi was extracted and immunoblotted utilizing anti-apoA-I Ab, as described in the Materials and Methods. Representative immunoblots of four mice per group are presented on the upper panel. Quantitation of the results (mean ± SEM; n = 5 per group) normalized relative to control apoE3 mice is presented on the bottom panel. White bars correspond to apoE3 mice, whereas black bars correspond to apoE4 mice. Two-way ANOVA revealed a significant effect for genotype (p = 0.003). *p<0.*05* for the post hoc comparison of control apoE4 and control apoE3 mice, and *p<0.*0*5 for the post hoc comparison of CS-6253-treated apoE4 and CS-6253 treated apoE3 mice. *(B) Native apoA-I ge*ls. Hippocampal extracts (*n* = 4 per group) from control and CS-6253 treated mice were electrophoresed on 4–16% gradient non-reducing native gel, as described in Materials and Methods. Representative immunoblots of three mice per group are presented and revealed that apoA-I migrated as two main bands of molecular highest of 25 and 45–50 kDa. The intensities of these native gel bands were not affected by CS-6253 and were higher in the apoE4 mice compared to the apoE3 mice.

We have previously shown that in the brain apoE4 is hypolipidated relative to apoE3 and that this effect is counteracted by peripheral application of CS-6253, which crosses the BBB and activates ABCA1 [25]. We presently examined the extent to which these effects are associated with changes in either the levels or extent of lipidation of brain apoA-I and apoJ. We first examined the levels of apoA-I in the hippocampus of apoE3- and apoE4-TR mice (Fig 5A). As can be seen, the total levels of apoA-I were higher in the apoE4 control mice compared to the control apoE3 mice, and were unaffected by the CS-6253 treatment (1.00 ± 0.06 versus 1.26 ± 0.06 for control apoE3 and control apoE4 mice, respectively, and 0.96 ± 0.09 versus 1.24 ± 0.08 for CS-6253-treated apoE3 and CS-6253-treated apoE4 mice, respectively). Two-way ANOVA revealed a significant effect for genotype (p = 0.003). Further *post hoc* analysis revealed that the levels of the control apoE4 mice were significantly higher than those of the corresponding apoE3 mice, and that the treated-apoE4 mice showed similarly higher levels of apoA-I compared to the treated apoE3 mice (p = 0.03 and p = 0.02, respectively). Native gel electrophoresis revealed that the apoA-I particles migrated as two bands, of apparent molecular weight of about 25kDa and 45–50 kDa, and that there were no differences in the size distribution of the apoA-I bands between the different groups. However, the intensities of the bands in the control and CS-6253-treated apoE4 mouse groups were higher than those of the corresponding apoE3 mice (Fig 5B), which is in accordance with the results obtained by measurements of the total apoA-I levels in the hippocampus (Fig 5A).

**Fig 6:**
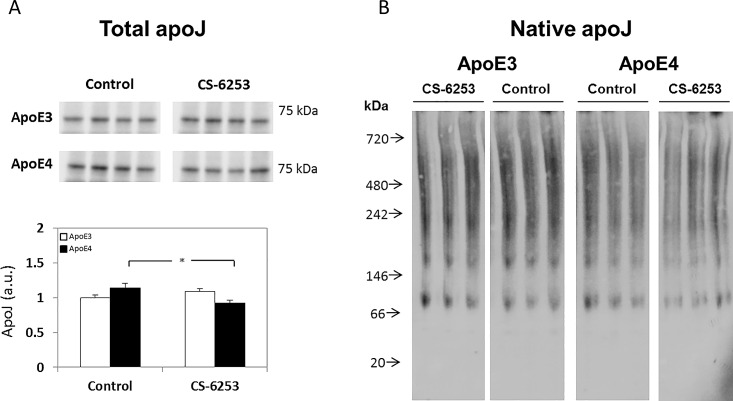
The effects of apoE genotype and CS-6253 on the levels and lipidation of brain apoJ in apoE3 and apoE4 mice. *(A) Total apoJ levels*. ApoE3 and apoE4 mice were injected i.p. with CS-6253 or PBS, after which their hippocampi was extracted and immunoblotted utilizing anti-apoJ Ab, as described in the Materials and Methods. Representative immunoblots of four mice per group are presented on the upper panel. Quantitation of the results (mean ± SEM; n = 8 per group) normalized relative to control apoE3 mice is presented on the bottom panel. White bars correspond to apoE3 mice, whereas black bars correspond to apoE4 mice. Two-way ANOVA revealed a significant effect for genotype x treatment (p = 0.006). *p<0.*01* for the post hoc comparison of control apoE4 and CS-6253-treated apoE4. *(B) Native apoJ gel*. Hippocampal extracts (*n* = 4 per group) from control and CS-6253 treated mice were electrophoresed on 4–16% gradient non-reducing native gel, as described in Materials and Methods section. Representative immunoblots of three mice per group are presented, showing that the pattern of apoJ was not affected by either apoE genotype or CS-6253 treatment, and appeared as particles ranging from about 100 to 700 kDa. In addition, the intensity of the staining of CS-6253 treated apoE4 mice was lower compared to the other groups.

Lastly, we assessed the effects of apoE-genotype and CS-6253 on the levels and extent of lipidation of brain apoJ. This revealed that the levels of apoJ in the control mice were slightly higher in the apoE4 mice compared to the corresponding apoE3 mice, and were decreased by CS-6253 in the apoE4 mice following treatment (1.00 ± 0.04 versus 1.13 ± 0.07 for control apoE3 and apoE4 mice, respectively, and 1.09 ± 0.04 versus 0.92 ± 0.04 for CS-6253-treated apoE3 and apoE4 mice, respectively; see Fig 6A). Two-way ANOVA revealed a significant effect for group x treatment (p = 0.006). *Post hoc* analysis of the results revealed that the levels of apoJ in the CS-6253-treated apoE4 mice was significantly lower than those of the control apoE4 mice (p = 0.005). Subsequent native apoJ immunoblotting revealed that the size distribution of the brain apoJ particles was similar in all mouse groups, and was unaffected by either genotype or treatment (Fig 6B). Importantly, the presently observed size distribution of the apoJ particles (~ 100 to 700 kDa, Fig 6B) was smaller than that of the corresponding brain apoE particles [~200 to 1000 kDa; [25]]. This is accordance with previous observations that in the brain apoE resides in in larger lipoprotein particles than apoJ [23]. The intensities of the brain apoJ staining in the native gel of the CS-6253-treated apoE4 mice was lower than those of the other groups as was observed in the SDS gel (Compare Fig 6A and 6B).

## Discussion

Brain apoE4 is hypolipidated relative to apoE3 [7, 25] and we have recently shown that treatment with the ABCA1 agonist CS-6253 reverses the hypolipidation of apoE4 and concomitantly counteracts the apoE4-driven brain pathology and cognitive impairments [25]. We presently investigated the extent to which these effects are related to plasma apoE4 and to the effects of apoE genotype and CS-6253 on brain and plasma apoJ and apoA-I levels and lipidation. The results thus obtained and the mechanisms underlying them are addressed below.

### (i) ApoE.

The apoE levels in the plasma of the control apoE4 mice were found to be lower than the corresponding levels in apoE3 mice (Fig 1A). Furthermore, there is a marked difference in the size of the apoE particles between the genotypes and their association with HDL-cholesterol, as depicted by the FPLC analysis (Figs 1B and 4B). These effects, namely the lower levels of apoE in apoE4 carriers and the difference in HDL-association, are in accordance with previous findings obtained from human studies in plasma and CSF [12, 36–38] and with sera and brain from different mouse models expressing human apoE [39–42].

In contrast to apoA-I, whose FPLC profile almost completely overlaps that of the HDL-cholesterol, the plasma of apoE co-elutes with both low-cholesterol containing particles (fractions 21–28) and high-cholesterol containing particles (fractions 28–35). This is in accordance with findings showing that the majority of HDL is associated with apoA-I, whereas only about 10% of HDL is associated with apoE [43, 44]. It is important to note that the control apoE4 profile is closely associated with the low-cholesterol containing particles, whereas the control apoE3 has a larger overlap with the HDL-cholesterol FPLC profile (compare Figs 1B and 4B which show a greater overlap between cholesterol and apoE3 following FPLC than between apoE4 and cholesterol). This suggests that the plasma apoE4 particles contain less cholesterol than the apoE3 particles. This is in accordance with the observation that apoE4 down regulates ABCA1 activity and cholesterol efflux by macrophages [45].

The major effect of CS-6253 treatment on the apoE4-containing particles is a shift from the low-cholesterol particles to the high-cholesterol containing particles, a shift that increases the overlap between the apoE4 FPLC profile and that of the HDL-cholesterol, and renders it similar to that of the apoE3 FPLC profile. This finding corresponds to the observation that in the brain, CS-6253 accelerates ABCA1-driven cholesterol efflux [46], thus shifting apoE4 particles to higher molecular weight species [25], which suggests increased lipidation of the apoE4 particles. Importantly, the plasma apoE3 particles are not affected by CS-6253.

With regards to the observation that apoE levels in the control apoE4 mice were lower compared to those in the corresponding apoE3 mice, it has been suggested that this is related to the decreased stability of apoE4 resulting in the susceptibility of apoE4 to proteolysis [12]. The enhanced vulnerability of apoE4 for degradation might relate to its lipidation state, causing it to be less stable [44]. Thus, treatment with CS-6253 which altered the lipid content of the apoE4 particle may contribute to its improved stability and increased levels.

Taken together, these findings suggest that like in the brain [25], the total levels of plasma apoE are lower in the apoE4 mice compared to those of the apoE3 mice and are less lipidated, and that CS-6253, which had no effect on either brain or plasma apoE3, has a pronounced effect on brain and plasma apoE4-lipid particles. In the plasma, this results in a shift of the apoE4 particles towards particles which co-elute with HDL-cholesterol, whereas in the brain the CS-6253 treatment increased the levels of apoE4 particles with decreased mobility [25].

It should be noted that while in the brain, apoE plays a major role in the distribution of lipids to cells, both during development and following injury [47, 48], in the periphery—it is mainly involved in reverse cholesterol transport to the liver [49, 50]. The distinct roles of apoE in the different pathways may reflect specific effects of apoE genotype and CS-6253 treatment on the apoE particles size and composition. Additional experiments, including measurements of the ratio of apoE to cholesterol and phospholipids and proteome analysis of these particles are needed to further the understanding of the effects of apoE genotype on the structure and size of the plasma lipoprotein particles.

ApoE4 is associated with cerebrovascular pathology and is a risk factor for vascular dementia [51, 52]. Preliminary histochemical findings suggest that, unlike the brain parenchyma [53], the cerebrovascular system of young 4-months old apoE4 mice is not markedly affected by apoE4 (in preparation), suggesting that the overall pathological effects of apoE4 in these mice and the associated protective effects of CS-6253 are largely brain parenchyma-driven. This assertion is also supported by the present observation that CS-6253 does not affect the levels of either cholesterol or triglycerides in the plasma (Fig 4).

### (ii) ApoA-I.

ApoE4 affects brain and plasma apoA-I levels in opposite directions. We presently report that the levels of plasma apoA-I are lower in the control apoE4 mice compared to the apoE3 mice, corresponding to the apoE levels, which were also lower in the control apoE4 mice. Similar results, depicting lower levels of apoA-I, were obtained from plasma of human apoE4 carriers [38]. Taken together, this suggests a possible regulatory link between apoA-I and apoE. The inter-relationship between apoA-I and apoE has been previously shown in the periphery, namely that apoA-I can induce the secretion of apoE, possibly through modulation of the apoE recycling process [54–56]. Alternatively, it has been shown that apoE3 supports the shift of apoA-I to HDL more so than apoE4, thus presumably stabilizing apoA-I [41, 57, 58]. Since apoE4 is associated with decreased levels of ABCA1, which are induced by CS-6253 treatment [25], it is possible that the apoE-apoA-I positive correlation under these condition is mediated via ABCA1 induction; however, it cannot be ruled out that this effect is due to direct cross-talk between the apolipoproteins. It remains to be determined whether in the plasma, apoA-I or apoE are the main target for ABCA1-induced lipidation, and which triggers the down-stream effects.

In contrast to the findings in the plasma, in the brain, apoA-I levels were elevated by apoE4 and were not affected by CS-6253 (Fig 5). The size of the brain apoA-I particles was smaller than those of the corresponding brain apoE and apoJ particles and is composed mainly of monomers and dimers (Fig 5B). This is in agreement with the finding that in the brain, apoE and apoA-I reside in different particles [59] and that apoA-I is less lipidated than apoE [14, 59]. The reason that apoA-I is less lipidated than apoE could be because apoA-I, unlike apoE, is synthesized peripherally and accumulates in the brain by crossing the BBB at the choroid plexus and that delipidated apoA-I accumulates readily in the brain [60].

ApoA-I plays a major role in peripheral cholesterol transport, and it is likely that it also plays an analogous role in the CNS [61–64]. However, data is sparse when addressing the interaction between apoA-I and apoE in the brain. The fact that the leading apolipoprotein in the periphery is apoA-I, while in the CNS it is apoE, may explain the differences in the levels of apoA-I in the plasma and in the brain of apoE4 mice. Accordingly, it is possible that the observed apoE4-driven increase in brain apoA-I is driven by a compensatory mechanism via which the increase in brain apoA-I levels counteracts lipid transport related deficits due to apoE4 and its decreased levels. Since the total brain levels of apoE4 are not affected by CS-6253 and remain lower than those of the corresponding apoE3 mice [25], such a mechanism is not expected to be affected by CS-6253, as indeed is the case. It is presumed that a different mechanism is at play in the plasma.

The observation that the levels of apoA-I in the plasma and brain are affected differently by apoE4 is in accordance with the finding that there is no correlation between the levels of apoA-I in the sera and CSF of individual subjects [65]. It still remains to be determined which plasma-related mechanism underlie the apoE4-driven decrease in plasma apoA-I and the reversal of this effect by CS-6253.

### (iii) ApoJ.

The levels of apoJ in the plasma of naïve apoE4 mice are significantly higher than those of the corresponding apoE3 mice, and this effect is reversed by treatment with CS-6253, which has no effect on the apoE3 mice (Fig 3A). A similar but less pronounced effect was observed in the brain (Fig 6A). The size and extent of lipidation of brain apoJ particles are smaller than those of apoE [i.e., brain apoJ particles range between ~200–700 kDa (Fig 5B), whereas the brain apoE particles ranged between ~200–1000 kDa; [25]], which is in accordance with previous findings [23, 35]. A similar effect was observed in the plasma, where the apoE particles eluted from the FPLC column at an earlier fraction than the apoJ particles, as previously described [compare Figs 1B and 3B [35]].

ApoJ serves both as a lipid transport protein and as a molecular chaperon [66–68]. The promoter region of the apoJ gene contains response elements for transcription factors involved in the cellular stress response and increased expression of apoJ is observed in several pathological conditions including AD, head trauma and epilepsy [for review, see [65]]. It is thus possible that the observed apoE4-driven increase in apoJ levels corresponds to a protective response to apoE4. This interpretation is supported by the observation that the anti-apoE4 protective effects of CS-6253 [25] are associated with complete abolition of the effects of apoE4 on apoJ (Figs 4 and 6). The cellular and molecular mechanisms underlying the elevation of apoJ by apoE4 are not known. However, the finding that the lipidation of apoJ is not affected by the CS-6253 treatment suggests that apoJ is less sensitive to ABCA1 modulation, as was previously suggested [13, 14]. This may indicate an indirect mechanism possibly mediated by apoE4-driven stress [69, 70].

In conclusion, this study shows that the levels of plasma apoE of apoE4 mice are substantially lower than those of the corresponding apoE3 mice and that this effect is associated with structural changes in the corresponding plasma apoE particles. These effects are accompanied by a compensatory increase in the levels of brain apoA-I and a significant increase in the levels of apoJ in the plasma. Treatment with the ABCA1 agonist CS-6253 which reverses the brain pathological effects of apoE4 and the associated cognitive impairments [25], reverses the effects of apoE4 on the lipidation and aggregation of plasma apoE4 without affecting the total corresponding apoE3 levels. This treatment abolishes the effects of apoE4 on plasma apoA-I and apoJ but not on the levels of brain apoA-I suggesting that the plasma apoJ levels are apoE4 stress responders whereas the levels of brain apoA-I are a compensatory response to the decreased levels of brain apoE4, which are unchanged by CS-6253 treatment.
